# Cardiac Cost During Submaximal Exercise as a Practical Monitoring Tool in French Standardbred Trotters: Short-Term Reproducibility of Non-Invasive Field-Derived Indicators

**DOI:** 10.3390/ani16111598

**Published:** 2026-05-24

**Authors:** Luc Poinsard, Claire Anson, Véronique Billat

**Affiliations:** 1 Movement, Balance, Performance, and Health Laboratory (EA 4445), Université de Pau et des Pays de l’Adour, 65000 Tarbes, France; lpoinsard@univ-pau.fr; 2 Horse Run Impulse Association, 77123 Noisy-sur-Ecole, France; claireanson@yahoo.fr; 3 Faculty of Sport Science, Université Évry Paris-Saclay, 91000 Évry-Courcouronnes, France

**Keywords:** horse, field exercise testing, reproducibility, heart rate recovery, cardiac cost, training monitoring

## Abstract

Routine monitoring in racehorses requires indicators that are reproducible and practical under real training conditions. This observational study evaluated the short-term reproducibility of cardiovascular and speed indicators in French Standardbred trotters, with a particular focus on cardiac cost (CC), defined as the ratio of heart rate to speed (beats·m^−1^). The full dataset comprised 483 sessions from 60 trotters and was used to describe age-related patterns. For reproducibility analyses, consecutive monitored sessions within the same horse were grouped into follow-up blocks when the interval between two successive sessions did not exceed 7 days. Only follow-up blocks containing at least three sessions were retained, resulting in 36 blocks, 126 sessions, and 18 horses. Each session included a warm-up, two 2000 m work blocks at increasing intensity, and recovery periods, while heart rate and speed were recorded using a Polar Team Pro system. Adjusted intraclass correlation coefficients indicated moderate reproducibility for CC during the first work block (CC B1: 0.67, 95% CI 0.48–0.78), heart rate recovery (HRR) after B1 (0.60, 0.40–0.73) and B2 (0.66, 0.47–0.78), and V150 (0.59, 0.39–0.73), whereas V180, recovery speed, and CC during B2 showed poor reproducibility. Reproducibility of CC B1 and HRR was preserved after adjustment for ambient temperature. In the full dataset, V200 increased with age, consistent with previous field-test literature. The minimal detectable change was 0.04 beats·m^−1^ for CC B1 and 26 bpm for HRR after B1. These findings suggest that CC B1, HRR, and V150 may be useful indicators for short-term monitoring, although results should be interpreted considering the single-yard design.

## Simple Summary

Monitoring how racehorses respond to exercise is important for adjusting training and detecting poor adaptation early. However, repeated measurements are only useful if they remain stable enough from one session to the next. This study examined which heart-related and speed-related indicators could be used for short-term monitoring in French Standardbred trotters during standardized training sessions. Each session included a warm-up followed by two exercise bouts at increasing intensity, while heart rate and speed were recorded with a wearable monitoring system. Data were collected from 483 sessions in 60 horses. Short-term repeatability was assessed in a subset of 126 sessions in 18 horses, selected by grouping repeated monitored sessions from the same horse when they were no more than 7 days apart. The most repeatable indicators were cardiac cost during the first exercise bout, heart rate measured 60 s after exercise, and the speed reached when heart rate first exceeded 150 beats per minute. By contrast, some other indicators, especially those derived from higher exercise intensities, were less stable from one session to the next. These results suggest that simple non-invasive measures recorded during routine training may help trainers and drivers monitor horses more effectively in the short term. The findings should nevertheless be interpreted with caution, because the study was based on horses from a single training yard.

## 1. Introduction

In the training of Standardbred trotters, monitoring the physiological response to exercise is central to performance management, because training decisions must balance workload progression with the early recognition of inadequate adaptation or poor performance. However, the challenge is not only to measure exercise responses, but also to determine whether observed changes exceed normal short-term variability and measurement error, and can therefore be interpreted as meaningful signs of training adaptation or altered physiological status. Standardized field exercise tests have long been used in trotters to provide objective physiological information under real training conditions [1,2]. Although such formal tests may not be performed routinely in all training yards, the increasing availability of wearable heart rate (HR) and speed-monitoring systems now makes it possible to derive comparable information from repeated standardized training sessions.

Field exercise testing in Standardbred trotters has historically relied on the combined measurement of speed, HR, and post-exercise blood lactate under standardized track conditions. In French trotters, these measurements have classically been used in standardized field-testing protocols to derive variables such as V4, the velocity at a blood lactate concentration of 4 mmol·L^−1^, and V200, the velocity at a HR of 200 beats·min^−1^ [1]. Population-based field studies showed that these derived variables were strongly influenced by age, training state, and performance level [3,4]. In these studies, both V4 and V200 increased with age and training state, and the best performers consistently showed the highest values, especially in younger horses [3,4].

However, although lactate-based indices remain central to the classical field-test literature, routine monitoring can also rely on non-invasive measures repeatedly obtained during ordinary training sessions. The widespread adoption of combined HR and GPS monitoring systems in professional equine training yards has made it increasingly feasible to derive fitness-related information from every standardized session, without the logistical constraints associated with blood sampling. In equine exercise testing, such information can be derived from cardiac responses, heart rate recovery (HRR), and speed at predefined HR thresholds. At lower exercise intensities, thresholds such as V150 and V160 have been used in addition to V200 [5,6]. Although the studies supporting these lower-threshold indicators were conducted in Warmblood sport horses rather than Standardbred trotters, they support the broader principle that field-derived fitness indices can be informative when training sessions are performed in a sufficiently standardized manner [6].

Alongside threshold-derived indices, integrative measures combining cardiovascular response and locomotor output may also be of practical interest for routine monitoring. Among these, cardiac cost (CC), defined as the ratio of HR to speed and expressed as beats per meter (beats·m^−1^), offers a conceptually simple and automatically derivable index of cardiovascular economy: a lower value means fewer heartbeats are needed to cover each meter of track, directly reflecting improved cardiovascular efficiency. Proposed in human exercise science as a sensitive index of cardiovascular strain during sustained effort [7], its physiological rationale transfers directly to equine locomotion, where HR and speed are both routinely available from field monitoring systems. Although its application in horses has not yet been formally evaluated, CC can be derived non-invasively from any session combining HR and speed recordings. Together with speed at predefined HR thresholds and HRR, an established marker of autonomic reactivation and cardiovascular fitness in horses [5], CC may therefore be of practical interest for routine monitoring, because all these indicators can be repeatedly extracted from standardized sessions without any invasive procedure.

Despite this body of work, the practical interpretation of repeated measurements within the same horse over a short time frame remains insufficiently documented. A test–retest study in Standardbred trotters showed good reproducibility of a standardized three-step field exercise test repeated after 7–9 days, with significant correlations for V200, V4, the velocity at a blood lactate concentration of 2 mmol·L^−1^ and the heart rate corresponding to a blood lactate concentration of 2 mmol·L^−1^ [2]. However, that study was based on relatively small groups, focused on classical thresholds derived from a dedicated step test, and did not specifically examine the short-term reproducibility of simpler cardiovascular and speed-related indicators extracted from repeated standardized training sessions. Crucially, it also predates the routine availability of continuous GPS and HR monitoring, which now makes session-to-session tracking of such indicators practically feasible in professional training yards. Furthermore, ambient temperature is known to affect cardiovascular responses during exercise in horses, independently of workload [8,9,10], yet its influence on the reproducibility of field-derived indicators has not been examined in Standardbred trotters, a critical gap for any monitoring system intended for year-round use. Thus, although field testing is well established for describing fitness, age-related differences, and performance level, it remains unclear which indicators are sufficiently stable to support session-to-session monitoring under the variable conditions of real training yards.

The aim of the present study was to determine which cardiovascular and speed-related indicators derived from a standardized field session are sufficiently reproducible to support short-term monitoring in French Standardbred trotters. A secondary aim was to describe age-related patterns in the full dataset to position the short-term monitoring subset within the established field-test literature. We hypothesized, first, that indicators reflecting cardiovascular load and post-exercise recovery would show higher short-term reproducibility than speed variables derived from higher HR thresholds, such as V180 and V200. These higher-threshold variables were expected to be more sensitive to day-to-day variation in exercise intensity, speed regulation, and the execution of the session. Second, consistent with the established literature, we predicted that V200 would increase significantly with age in the full dataset, thereby confirming that the present study population behaves in line with previously described French Standardbred cohorts and lending interpretive context to the reproducibility analyses.

## 2. Materials and Methods

### 2.1. Study Design and Data Source

This observational study was based on routinely collected training and monitoring data from French Standardbred trotters trained in a professional yard located at the Domaine de Grosbois (Marolles-en-Brie, Val-de-Marne, France). Data were collected as part of routine stable monitoring, and the sample size therefore reflects data availability rather than a predetermined power calculation. Monitored sessions were not scheduled by the investigators at fixed weekly intervals or specifically in relation to race entries. Instead, they corresponded to routine standardized training sessions performed at the trainer’s discretion within the usual professional training program.

To avoid ambiguity, the term “training session” refers to one monitored standardized exercise session performed during routine training. The term “follow-up block” refers to a group of consecutive monitored sessions from the same horse used for the short-term reproducibility analysis. The within-session exercise structure, including the two work blocks referred to as B1 and B2, is described in Section 2.3.

The full dataset available for the present analyses comprised 483 standardized sessions recorded in 60 trotters. These sessions were considered standardized because they followed the same overall structure, including two 2000 m work blocks separated by a fixed 4 min active recovery period, and predefined target speeds adapted according to age and training period. Analyses were conducted at two levels. First, the full dataset was used to describe age-related patterns in selected field-derived variables. Second, a short-term monitoring subset was constructed to assess the reproducibility and short-term stability of physiological and performance indicators across repeated sessions performed under comparable temporal conditions.

### 2.2. Horses and Construction of Short-Term Follow-Up Blocks

Short-term follow-up blocks were constructed by grouping consecutive monitored sessions within the same horse when the interval between two successive monitored sessions did not exceed 7 days. Thus, the 7-day criterion applied to the gap between successive sessions, not necessarily to the total duration of the follow-up block, which could be longer when a block contained more than two sessions. This design was intended to capture periods of close monitoring while limiting temporal heterogeneity between repeated measurements.

Between monitored sessions, horses remained in their usual professional training and management program under the responsibility of the trainer. Intervening activities, such as additional exercise sessions or rest days, were not prescribed by the study protocol and were not systematically recorded in the dataset.

Several candidate maximum inter-session intervals were explored during dataset construction, including 4, 7, and 15 days. A 7-day threshold was retained for the main analysis because it provided the most appropriate balance between short-term comparability of repeated measurements and retention of a sufficient number of horses, follow-up blocks, and sessions for reproducibility analyses. This interval was also considered consistent with the 7–9-day retest interval previously used in Standardbred field-testing studies [2]. Sensitivity analyses using alternative follow-up block definitions are summarized in Supplementary Table S1A,B. Only follow-up blocks containing at least three sessions were retained for analysis.

The descriptive characteristics of the full dataset and of the short-term monitoring subset are summarized in Table 1. Time in training was not systematically recorded in the routine monitoring dataset and is therefore not reported.

### 2.3. Standardized Field Exercise Session

Each monitored training session followed the same overall structure and was repeatedly performed during routine training. Before entering the professional training yard, young horses were prepared in a partner breeding and pre-training yard associated with the trainer. After breaking-in, they progressively performed training sessions resembling the standardized work later used in the professional yard, with distances, durations, and speeds adapted according to age and training level. Consequently, all horses, including the youngest 2-year-old horses, were already accustomed to the general structure and demands of this type of standardized training session before inclusion in the routine monitoring dataset.

Each session included an approximately 10 min warm-up, ending with a brief acceleration bout of about 30 s, followed by two 2000 m exercise bouts performed at increasing target speeds. To avoid confusion with the follow-up blocks used for reproducibility analyses, these within-session exercise bouts are referred to throughout the manuscript as work blocks B1 and B2.

B1 and B2 were separated by a fixed 4 min active recovery period. The duration of this between-block recovery period was standardized across sessions, whereas its pace was not strictly imposed and could vary according to age and the trainer’s judgment. After B2, no additional standardized 4 min recovery period was imposed. The post-B2 recovery variables were therefore extracted during the cool-down and return-to-stable phase.

Exercise intensity during B1 and B2 was adapted according to age and training period using predefined target speeds within the standardized training framework (Table 2).

These target speeds were not individualized according to formal physiological testing or each horse’s measured maximal capacity. Instead, they reflected the standardized training targets used in the yard for horses of a given age and training period. Consequently, the same nominal target speed could represent slightly different relative intensities between horses, which is inherent to routine field-monitoring conditions.

In the present study, the monitored sessions corresponded to the second and third stages of the framework described by Demonceau and Auvinet (1992) [11], which are here referred to as B1 and B2. Accordingly, the present analyses were restricted to these two work blocks and their associated recovery periods.

### 2.4. Instrumentation and Data Acquisition

HR and movement-derived variables were recorded continuously during training using a Polar Team Pro system (Polar Electro Oy, Kempele, Finland), fitted with a Polar Equine Belt. To our knowledge, direct peer-reviewed validation of this exact configuration in horses has not yet been reported. However, convergent evidence supports the use of its main components for field monitoring. For HR acquisition, comparable Polar chest-strap systems have shown good agreement with ECG-derived measurements in horses under stationary, groundwork, or exercising conditions, although artifact handling remains important and agreement is generally stronger for HR than for more complex HR variability metrics [12,13,14]. For speed and distance acquisition, the 10 Hz Global Navigation Satellite System (GNSS) component of the Polar Team Pro has shown acceptable validity and reliability in sports settings [15,16]. The system was therefore used here as a practical field-monitoring device for repeated standardized sessions, with subsequent signal cleaning applied before derivation of HR- and speed-related indicators [12,14]. After each session, recordings were uploaded to the Polar Team Pro platform and exported for subsequent analysis.

### 2.5. Signal Processing and Data Cleaning

Prior to variable derivation, HR and GNSS recordings were reviewed and cleaned to remove obvious artifacts before segmentation of the standardized session. For HR data, observations with values <25 or >240 beats·min^−1^ were treated as implausible and removed. In addition, abrupt HR changes >7 beats·min^−1^ between consecutive 1 s observations were considered artifactual and excluded. This threshold was used as a pragmatic rule-based artefact filter to remove isolated non-physiological spikes while preserving the overall exercise HR response. The Polar Team Pro system was not used as a diagnostic ECG device, and exercise-induced arrhythmias could therefore not be formally assessed. Visual inspection of the recordings did not reveal sustained erratic HR patterns incompatible with reliable analysis during the analyzed work blocks or recovery periods.

For GNSS data, quality control targeted unrealistic displacement spikes and non-physiological speed values using rule-based plausibility checks applied at the 10 Hz epoch level, including removal of observations with instantaneous speed > 15 m·s^−1^ and/or point-to-point displacement > 3 m between consecutive 0.1 s samples. Invalid observations were removed before variable derivation. When recordings showed major signal loss or clearly erratic traces, the affected data were not retained for analyses involving the corresponding variables. Cleaned HR and speed recordings were then segmented according to the predefined structure of each session, including the two work blocks and their associated recovery periods.

### 2.6. Derived Variables

Analyses were restricted to indicators considered directly relevant for routine trainer monitoring during repeated standardized sessions. V150, V180 and V200 were defined as the speed corresponding to the first valid occurrence at which HR reached or exceeded 150, 180 and 200 beats·min^−1^, respectively, based on the cleaned HR and speed recordings from the two work blocks. A threshold crossing was considered valid only if HR remained at or above the target threshold for at least 10 consecutive seconds. This rule was defined a priori to reduce misclassification of isolated transient fluctuations or artefactual spikes as true threshold crossings. Requiring sustained threshold attainment over 10 consecutive seconds was considered a pragmatic compromise between robustness to signal noise and preservation of field applicability. For each threshold, the retained value corresponded to the earliest valid occurrence across the two work blocks, whether observed in B1 or B2.

Additional variables of interest were mean speed during the first and second work blocks, CC during each work block, and HR and speed measured 60 s after the end of each work block as indices of short-term recovery. In the present study, HRR refers to the absolute HR value recorded 60 s after the end of each work block. HRR was defined as the instantaneous value recorded at that time point. Given the consistently descending HR trajectory observed during active recovery at the intensities studied, a single time-point measure was considered appropriate. This 60 s time point was selected for practical and protocol-related reasons. The standardized session included a 4 min active recovery period between B1 and B2, so longer recovery indices such as 10- or 12 min HRR could not be obtained after B1 without modifying the routine training session. The 60 s time point also allowed recovery to be assessed after both work blocks within the same field protocol, whereas a 10- or 12 min value would mainly have reflected end-of-session recovery and would have been more dependent on the cool-down and return-to-stable conditions. Therefore, 60 s HRR was selected as an early recovery indicator suitable for repeated routine monitoring within this two-block protocol, rather than as a replacement for longer recovery measures such as the 10- or 12 min HRR values used in other field-testing contexts, by practitioners, or in studies such as Ringmark et al. [17].

CC for each work block was calculated as [7]:

(1) $$CC\;({beats\cdot m}^{-1})=\frac{\bar{HR}{(beats\cdot min}^{-1})}{\bar{v}{(m\cdot min}^{-1})}$$

where $\bar{HR}$ represents the mean HR over the work block and $\bar{v}$ the mean speed over the same period, with speed converted from km·h^−1^ to m·min^−1^ by multiplying by 1000/60.

These variables were selected because they represent simple cardiovascular and speed-related field measures that can be repeatedly derived from standardized routine training sessions.

### 2.7. Meteorological Covariates

Hourly temperature data were obtained from Météo-France using the nearest available station to the Domaine de Grosbois (Saint-Maur-des-Fossés, Val-de-Marne, France). For each session, temperature was averaged across the corresponding hourly records, with each value weighted according to the duration of temporal overlap between the session and the hourly meteorological interval. Mean session temperature ranged from −1.6 to 23.1 °C across the monitoring period (mean ± SD: 12.0 ± 5.3 °C).

### 2.8. Ethical Considerations

This study was based on non-invasive monitoring performed during routine training sessions in a professional training yard. Horses were accustomed to regular handling and to the use of training equipment. Data were collected using an externally worn Polar Equine Belt and did not involve any invasive procedure or manipulation expected to cause pain, suffering, distress, or lasting harm. The trainer responsible for the yard agreed to the use of routinely collected monitoring data for research purposes, and the horse owners were informed of this research use and provided their consent. Under French regulations governing the use of animals for scientific purposes, acts performed in agricultural holdings for non-experimental purposes, as well as procedures not liable to cause pain, suffering, distress, or lasting harm equivalent to or greater than that caused by the introduction of a needle in accordance with good veterinary practice, fall outside the scope of regulated experimental procedures (Code rural et de la Pêche maritime, Articles R. 214-88 and R. 214-89). Accordingly, no formal project authorization or ethical review was sought for this observational study.

### 2.9. Statistical Analysis

All analyses were performed in R (version 4.5.2, R Core Team, Vienna, Austria) within RStudio (version 2026.01.1+403, Posit Software, PBC, Boston, MA, USA). Linear mixed-effects models were fitted using the lme4 (version 2.0.1) and lmerTest (version 3.2.1) packages [18,19].

The primary objective was to assess the short-term reproducibility of repeated measurements within follow-up blocks. Within-follow-up-block reproducibility was assessed using intraclass correlation coefficients (ICCs) derived from linear mixed-effects models fitted with restricted maximum likelihood estimation, with follow-up block included as a random intercept (1|block_uid), in accordance with current methodological recommendations for reliability studies [20]. For each variable, crude ICCs were first estimated from intercept-only models, corresponding to one-way random-effects single-measure ICCs, ICC(1,1) in the framework of Koo and Li [20], and calculated as the proportion of total variance attributable to differences between follow-up blocks according to the variance-components formula:

(2) $$ICC=\frac{\sigma^{2}block}{\left(\sigma^{2}block+\;\sigma^{2}residual\right)}$$

where $\sigma^{2}block$ represents the between-block variance and $\sigma^{2}residual$ the residual within-block variance. Adjusted ICCs were estimated from models additionally including age, sex, and mean session temperature as fixed effects, using the same variance-components formula applied to the variance components of the adjusted model. These are interpreted as covariate-adjusted proportions of variance attributable to between-block differences, and do not correspond directly to a standard ICC type in the Koo and Li framework. Ninety-five percent confidence intervals for all ICC estimates were obtained by parametric bootstrap (1000 iterations). ICC values were interpreted according to Koo and Li as poor (<0.50), moderate (0.50–0.75), good (0.75–0.90), or excellent (>0.90) [20]. Because ambient temperature may influence cardiovascular responses during exercise, additional adjusted analyses were also performed to assess whether the main reproducibility findings remained robust after accounting for mean session temperature. For this purpose, ICC estimates adjusted for age and sex only were compared with ICC estimates additionally adjusted for mean session temperature.

To improve practical interpretability beyond relative reliability alone, absolute measurement error was also summarized for the adjusted models using the standard error of measurement (SEM) and the minimal detectable change at the 95% confidence level (MDC95), calculated as 1.96 × √2 × SEM [21,22]. In the present mixed-model framework, SEM was estimated as the square root of the residual variance. These indices are reported in Supplementary Table S2.

To evaluate average change over the monitoring period, the first and last session of each follow-up block were compared for each variable. Because these comparisons involved paired observations within follow-up block, analyses were based on the distribution of paired differences. Normality of paired differences was assessed using the Shapiro–Wilk test. Paired *t*-tests were used when the normality assumption was considered acceptable, whereas Wilcoxon signed-rank tests were preferred otherwise.

Because HR measured 60 s after exercise may partly depend on active recovery speed, additional sensitivity analyses were performed for HR recovery after B1 and B2. Linear mixed-effects models were fitted with HR recovery as the outcome and recovery speed measured at the same time point as the main predictor, while adjusting for age, sex, and mean session temperature. Horse and follow-up block were included as random intercepts. Adjusted ICCs for HRR were also recalculated after inclusion of recovery speed as a covariate, to assess whether the reproducibility of HRR was explained by differences in active recovery speed.

For the secondary objective, age-related associations in the full dataset were examined using the same model specification, with age, sex, and mean session temperature as fixed effects and horse as a random intercept.

## 3. Results

### 3.1. Within-Follow-Up-Block Reproducibility of Selected Indicators

**Figure 1:**
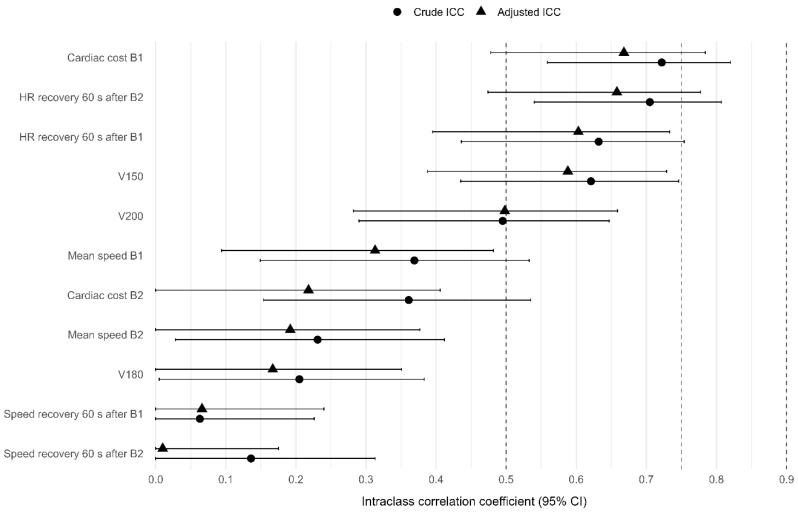
Crude and adjusted intraclass correlation coefficients (ICCs) with 95% confidence intervals for the selected cardiovascular and speed-related indicators. Crude ICCs were estimated from intercept-only mixed-effects models with follow-up block as a random intercept. Adjusted ICCs were estimated from analogous models including age, sex, and mean session temperature as fixed effects. Dashed horizontal lines indicate the interpretation thresholds proposed by Koo and Li [20]: poor (<0.50), moderate (0.50–0.75), good (0.75–0.90), and excellent (>0.90). CI: confidence interval; V150, V180, V200: speed at a heart rate of 150, 180, and 200 beats·min^−1^, respectively; HR: heart rate; B1, B2: first and second work blocks, respectively.

Crude and adjusted ICC estimates with 95% confidence intervals are shown in Figure 1.

The corresponding numerical values, together with SEM and MDC95, are reported in Supplementary Table S2. According to the interpretation framework proposed by Koo and Li [20], adjusted ICCs indicated moderate reproducibility for V150, CC during B1, and HRR 60 s after both B1 and B2, whereas the remaining indicators showed poor reproducibility. The highest adjusted ICCs were observed for CC during B1 (ICC = 0.67, 95% CI 0.48–0.78), HRR 60 s after B2 (ICC = 0.66, 95% CI 0.47–0.78), HRR 60 s after B1 (ICC = 0.60, 95% CI 0.39–0.73), and V150 (ICC = 0.59, 95% CI 0.39–0.73). By contrast, V180, speed measured at 60 s during recovery after both B1 and B2, and CC during B2 showed low reproducibility. V200 remained borderline, with an adjusted ICC of 0.50 (95% CI 0.28–0.66).

Adjustment for mean session temperature had little impact on the ICC estimates of the main indicators. ICC values changed by less than 0.01 for CC during B1, HRR after B1, HRR after B2, and V150, and the ranking of the most reproducible indicators remained unchanged. By contrast, mean speed during B1 and speed recovery after B2 showed larger decreases in ICC after temperature adjustment (−0.08 and −0.09, respectively), suggesting greater sensitivity of these variables to session-level ambient conditions; however, given their poor reproducibility across all model specifications, they were not retained as primary monitoring candidates. The full comparison of ICC estimates obtained with age and sex adjustment only versus additional adjustment for mean session temperature is provided in Supplementary Table S4.

Descriptive statistics for the selected indicators in the 7-day short-term monitoring subset are provided in Supplementary Table S3 and sensitivity analyses using alternative maximum inter-session intervals are summarized in Supplementary Table S1A,B.

### 3.2. Sensitivity Analysis for 60 s Heart Rate Recovery

Because HR measured 60 s after exercise may partly depend on active recovery speed, additional sensitivity analyses were performed including recovery speed at 60 s as a covariate. As expected, higher recovery speed was associated with higher HR at 60 s after both B1 (β = 1.62 bpm per km·h^−1^, *p* < 0.001) and B2 (β = 0.86 bpm per km/h, *p* < 0.001). However, the reproducibility of HRR remained moderate after accounting for recovery speed, with adjusted ICCs of 0.69 (95% CI 0.51–0.80) for B1 and 0.68 (95% CI 0.50–0.79) for B2. These values were slightly higher than the ICC estimates obtained without recovery speed as a covariate (B1: 0.60; B2: 0.66). By contrast, speed measured at the same recovery time point showed very low reproducibility across all model specifications.

### 3.3. Complementary First-to-Last Session Comparisons Within Follow-Up Blocks

First-to-last session comparisons within follow-up blocks are presented in Table 3.

Among the selected indicators, significant differences between the first and last session of the follow-up block were identified for V180 and mean speed during B2 only, both showing a positive mean change. All other variables showed no significant difference between the first and last session of the block.

### 3.4. Age-Related Patterns in the Full Dataset

In the full dataset, the median number of sessions per horse was 4.0 (IQR 2.0–9.0). V200 increased significantly with age (estimate 0.4 km·h^−1^ per year, *p* < 0.001), whereas the association with age was weaker for V180 (*p* = 0.07) and was not significant for V150 (*p* = 0.22). Age was also negatively associated with CC in both work blocks, with HRR measured 60 s after exercise, and with speed measured at the same recovery time point. These age-related associations are summarized in Table 4.

## 4. Discussion

The present study aimed to identify which cardiovascular and speed-related indicators derived from a standardized training session were sufficiently reproducible to support short-term monitoring in French Standardbred trotters. The main findings were that CC during B1, HRR measured 60 s after both B1 and B2, and V150 showed the most favorable short-term reproducibility, whereas speed variables derived from higher HR thresholds, recovery speed, and CC during B2 showed lower reproducibility. In parallel, the secondary analysis confirmed that V200 increased significantly with age in the full dataset, in line with the established field-test literature in Standardbred trotters. These findings partly support our initial hypothesis. Indicators reflecting cardiovascular load and post-exercise recovery were among the most reproducible variables within short-term follow-up blocks, as predicted. However, the hypothesis was only partially confirmed, because CC showed markedly different reproducibility between B1 and B2, and V150 performed comparably to the best cardiovascular indicators. This unexpected finding warrants specific discussion.

### 4.1. Reproducibility of Cardiovascular Load and Recovery Indicators

The moderate reproducibility of CC during B1 and of HRR after both B1 and B2 supports the practical value of these indicators for routine monitoring. CC integrates cardiovascular demand relative to locomotor output and has been proposed in human exercise science as a sensitive index of cardiovascular strain during sustained effort [7]. Its relatively stable behavior during B1 in the present study is consistent with the idea that, at lower exercise intensities, the cardiovascular response to a standardized submaximal effort is more reproducible from session to session, as intra-individual variation in HR at a given submaximal speed tends to be limited when conditions are comparable [6].

HRR after exercise has long been recognized as an informative indicator of cardiovascular fitness and autonomic function in horses [5], and this broader physiological and practical relevance has been emphasized in a recent comparative review of the human and equine athlete highlighting the shared autonomic mechanisms underlying post-exercise cardiac deceleration across species [23]. Its utility for routine monitoring in Standardbred trotters has been supported by Ringmark et al., who showed that HRR was sensitive to short-term differences in training load even when classical lactate-derived indices such as V4 were unaffected [17]. In the present study, HRR was analyzed as an absolute HR value measured 60 s after each work block. Because no maximal exercise test was performed, true maximal heart rate was not available, and exercise intensity could not be expressed as a percentage of individual HRmax. This should be considered when interpreting HRR, since recovery kinetics may be influenced by the relative intensity reached during the preceding exercise bout. Nevertheless, the moderate reproducibility observed here for HRR after both B1 and B2, even after adjustment for active recovery speed, further reinforces this interpretation and suggests that HRR captures a meaningful component of cardiovascular response that is relatively stable within short monitoring periods.

Adjustment for mean session temperature had little effect on the ICC estimates of the main indicators and did not modify their ranking. This finding should not be interpreted as evidence that ambient temperature had no physiological effect on cardiovascular responses during exercise [8,9,10]. ICCs quantify relative reproducibility, and temperature may still have influenced absolute HR, speed, or recovery values. In addition, only ambient temperature was systematically available, whereas relative humidity, wind, solar radiation, and track condition were not recorded. These unmeasured factors may have influenced thermoregulatory demand, locomotion, and cardiovascular responses, and may partly explain residual between-session variability.

Taken together, cardiac cost B1 and HRR emerge as informative non-invasive indicators for session-to-session monitoring in this population, combining moderate reproducibility and physiological interpretability, while remaining subject to environmental and field-condition influences.

### 4.2. The Cardiac Cost Asymmetry Between B1 and B2

One of the more notable findings was the marked asymmetry in reproducibility between CC during B1 (adjusted ICC = 0.67) and during B2 (adjusted ICC = 0.23). Because CC is defined identically across both work blocks, this difference cannot be attributed to the indicator itself but must reflect differences in the exercise conditions between B1 and B2. B2 was performed at a higher target speed than B1, placing horses at higher relative exercise intensities. At higher exercise intensities, HR responses are known to be more variable and more sensitive to day-to-day fluctuations in fatigue, motivation, driver-dependent speed regulation, and cumulative cardiovascular load accumulated during B1 [24]. Cardiovascular drift, the progressive increase in HR observed during sustained submaximal exercise at constant workload, documented in horses under controlled conditions [10], may further amplify HR variability during B2, as the cardiovascular state at the start of B2 is partly influenced by the residual load from B1, which may itself vary between sessions. Together, these factors plausibly explain why CC during B2 was considerably less reproducible than during B1, even though both were derived from the same standardized training structure.

From a monitoring perspective, the higher reproducibility of CC during B1 suggests that this first submaximal work block may provide a more stable reference point than B2 for repeated session-to-session comparisons. The practical implementation of this indicator is discussed further in Section 4.7.

### 4.3. Reproducibility of Threshold-Derived Speed Indicators

V150 showed moderate reproducibility and performed comparably to the best cardiovascular indicators, whereas V180 remained clearly less reproducible, and V200 was only borderline. This gradient across thresholds suggests that lower-threshold speed indicators may be more stable under routine field conditions than higher-threshold ones. A plausible explanation is that lower HR thresholds are reached more consistently across sessions and at exercise intensities where the HR–speed relationship is less steep, so that small between-session differences in speed regulation or cardiovascular state translate into smaller differences in the derived speed value. By contrast, at intensities approaching HR180 or HR200, the relationship between HR and speed becomes steeper and more sensitive to fatigue, speed variation, and the progression of effort between B1 and B2 [5,24]. In addition, V150 is more likely to be attained earlier in the standardized session, often during B1, whereas V180 and especially V200 may depend more strongly on the sequential progression from B1 to B2. It should also be noted that threshold speeds in the present study were derived from the first valid threshold crossing across the two sequential work blocks rather than from a model-based reconstruction of the full HR–speed relationship, a pragmatic approach that is well-suited to routine monitoring but one that may introduce additional session-to-session variation at higher thresholds where the crossing point depends more strongly on the exact progression of effort.

Because V150 is reached at a lower submaximal intensity, it was more consistently attainable across horses and sessions, which may partly explain its higher reproducibility in the present study. However, its favorable short-term reproducibility should not be interpreted as evidence that V150 is superior to higher-intensity or lactate-based indices for assessing fitness or performance capacity. In French Standardbred trotters, classical variables such as V4 and V200 have been shown to vary with age, training state, and performance level [3,4]. Therefore, V150 should be interpreted as a practical low-intensity indicator for routine non-invasive monitoring, whereas higher-intensity and lactate-based indices remain important for more complete physiological or performance assessment.

### 4.4. Sensitivity Analysis for Heart Rate Recovery

The sensitivity analysis confirmed that active recovery speed was positively associated with HR measured 60 s after both B1 and B2, as expected from basic physiological principles. However, the moderate reproducibility of HRR was preserved after adjustment for recovery speed—with adjusted ICCs of 0.69 (95% CI 0.51–0.80) for B1 and 0.68 (95% CI 0.50–0.79) for B2, slightly higher than without this adjustment (B1: 0.60; B2: 0.66)—indicating that the usefulness of HRR cannot be reduced to variation in active recovery pacing alone. This finding is consistent with the broader literature in which post-exercise HR is regarded as a reflection of autonomic reactivation and cardiovascular fitness rather than simply a function of residual locomotor activity [5,17].

While keeping recovery conditions as standardized as possible remains desirable, moderate variation in active recovery speed does not fully explain session-to-session differences in HRR values, which reinforces its relevance as a post-session monitoring indicator independent of pacing behavior.

### 4.5. Age-Related Patterns and Positioning Within the Literature

The age-related analyses in the full dataset confirm that V200 increased significantly with age, which is consistent with well-established findings in French Standardbred trotters [3,4] and with more recent work highlighting V200 as one of the more useful fitness-related parameters in equine performance assessment [25]. In contrast, the associations with age were weaker or absent for V150 and V180. This pattern is physiologically coherent. V200 is attained at higher relative exercise intensities and is therefore more sensitive to the cardiovascular and metabolic adaptations that accumulate with age and progressive training, whereas lower thresholds may be reached at intensities that are insufficiently discriminating to capture fitness-related differences across age groups. The negative associations of age with CC and post-exercise HR are also consistent with improved cardiovascular efficiency and faster autonomic recovery as horses mature and adapt to training, a pattern that has been documented in equine training studies examining HR responses across different ages and fitness levels [10,17]. Taken together, these age-related findings suggest that the short-term monitoring subset was broadly consistent with the physiological patterns observed in the full dataset and with the established French Standardbred field-test literature, which strengthens the interpretive context for the reproducibility analyses.

### 4.6. First-to-Last Session Comparisons and Their Relationship with ICC Results

The complementary first-to-last comparisons within follow-up blocks address a distinct question from the ICC analyses and should be interpreted accordingly. ICC assesses the relative stability of repeated measurements within follow-up blocks by determining whether the rank ordering of horses is preserved across sessions, whereas first-to-last comparisons test whether a variable changes on average over time. Accordingly, a variable may show a significant mean change while still displaying poor reproducibility at the individual level, as observed here for V180. Only V180 and mean speed during B2 showed significant positive mean changes between the first and last session of the follow-up block, whereas all other indicators, including cardiac cost B1 and HRR, showed no systematic directional change. One possible interpretation is that V180 changed heterogeneously across horses, with some animals improving more than others over the short monitoring period, thereby producing a positive average change without strong rank-order stability across repeated sessions. The observed positive mean changes may reflect short-term training adaptation, increased familiarity with the protocol, or natural variation within a population of young horses in active training, although distinguishing between these explanations was beyond the scope of the present study.

Sensitivity analyses on follow-up block definition further supported the choice of a 7-day maximum inter-session interval as a reasonable compromise. Compared with a 4-day threshold, the 7-day definition retained a substantially larger monitoring subset while preserving broadly similar reproducibility patterns. By contrast, extending the maximum interval to 15 days increased sample size but was associated with lower adjusted ICCs for several key indicators and with longer follow-up block durations, thereby reducing the temporal comparability of repeated measurements [2].

### 4.7. Practical Implications

The present findings support a simple, operationally feasible monitoring framework for professional trotting yards, structured around three non-invasive indicators derived from standardized routine training sessions: CC during B1, HRR after both work blocks, and V150. These indicators are not intended to replace formal physiological testing, but to provide trainers with practical tools for monitoring the horse’s response more frequently between dedicated evaluations.

CC during B1 is the central element of this framework because it is derived from the first submaximal work block, before the horse reaches the highest training intensities of the session. In the current workflow, however, CC B1 was calculated after data export and should therefore be considered primarily as a post-session indicator for reviewing the horse’s response and guiding subsequent training decisions. Its potential use as a true mid-session indicator would require further technological development, such as automated real-time calculation and display of cardiac cost from live HR and speed data. This appears technically feasible because HR and speed are already available in real time in many wearable monitoring systems, but the derived CC value is not yet routinely displayed to the trainer or driver.

Session-to-session comparisons using the MDC95 threshold of 0.04 beats·m^−1^, corresponding to approximately 21 bpm at 35 km·h^−1^, may help identify sessions in which the horse did not respond as expected. However, this interpretation currently remains more applicable to post-session review than to immediate in-session decision-making. HRR after both work blocks complements cardiac cost as a post-session indicator of autonomic recovery and cardiovascular adaptation, with MDC95 thresholds of 26 bpm after B1 and 22 bpm after B2. V150 provides an additional speed-based dimension when threshold attainability is consistent.

Importantly, practitioners must be clearly informed that these MDC95 thresholds are not trivial. For HRR, the required change represents approximately 20% of the mean value, meaning that small fluctuations within this range should not be over-interpreted as true physiological signals. The moderate ICC values obtained here reflect population-level relative reliability rather than high individual-level precision, a distinction that is essential for responsible implementation [21,22].

Compared with a dedicated exercise test [2], the indicators evaluated here can be derived from routine training sessions and may therefore help trainers monitor the horse’s response more frequently between formal evaluations. This practical advantage should not be interpreted as meaning that HR- and speed-based indicators can replace lactate measurements, veterinary assessment, or standardized incremental exercise testing. Rather, they should be viewed as routine screening and monitoring tools that can help identify unusual responses during everyday training. When persistent or abnormal deviations are observed, more detailed investigation is warranted, and standardized incremental exercise testing with lactate assessment may provide substantially more physiological and clinical information, particularly when the objective is to diagnose performance limitations or detect subtle changes in physical condition.

Its integration into current Polar Team Pro or equivalent data pipelines could be partially automated, reducing the operational burden on yard personnel and making evidence-based monitoring accessible in routine professional settings. The growing literature on non-invasive monitoring in racehorses, including studies in Thoroughbreds, has focused on training load quantification and performance prediction [26,27,28], supports this broader shift toward session-derived physiological indicators. The present study extends this body of work by providing reproducibility benchmarks specifically for French Standardbred trotters under routine training conditions.

From a clinical perspective, future prospective studies should determine whether deviations in CC B1, HRR, or V150 beyond their respective MDC95 thresholds are associated with subsequent performance- or welfare-related outcomes. Such analyses should not rely solely on raw race results, because race performance may be influenced by strategic entry decisions, race conditions, handicap rules, opposition level, driver or jockey tactics, and the specific objective of a given race. More informative outcomes may include adjusted performance indicators, poor-performance episodes, veterinary findings, lameness, respiratory disease, or other welfare-relevant events. Such work would be needed before these indicators can be interpreted as clinical warning markers or used to predict problems before they affect welfare or performance. The present study should therefore be considered a reproducibility step toward clinically meaningful monitoring, rather than a validation of diagnostic or prognostic thresholds.

### 4.8. Limitations

Several limitations should be acknowledged. First, the study was based on data from a single professional yard, and the short-term reproducibility subset remained modest in size (n = 18 horses, 36 blocks), which is reflected in the width of some confidence intervals. Generalizability to other training contexts, track surfaces, and horse populations therefore cannot be assumed. In addition, time in training was not systematically recorded in the routine monitoring dataset and could therefore not be included in the descriptive analyses or statistical models. Consequently, age should be interpreted only as an available descriptor of maturation and training exposure, rather than as a direct measure of individual training history. Second, the exact Polar Team Pro/equine-belt configuration used here has not been directly validated in horses, although convergent evidence supports the use of comparable Polar systems for HR monitoring and the GNSS component for speed-related measurements. In particular, this configuration has not been directly validated at very high heart rates, especially around or above 200 beats·min^−1^. This limitation should be considered when interpreting high-threshold variables such as V200, which may be more sensitive to measurement error than lower-intensity indicators such as V150, cardiac cost during B1, and HRR. Third, because no maximal exercise test was performed, true maximal heart rate was not available, and the relative intensity of each work block could not be expressed as a percentage of individual HRmax. This may be relevant for HRR interpretation because recovery kinetics can be influenced by the intensity reached during the preceding exercise bout. Fourth, the present study focused deliberately on non-invasive routine indicators and did not include lactate measurements. Therefore, the findings should not be interpreted as replacing classical V4-based or standardized incremental exercise testing. Lactate assessment and formal exercise testing remain important when the objective is to investigate performance limitation, detect subtle changes in physical condition, or support veterinary decision-making. Fifth, although temperature was the only environmental variable for which data were systematically available, other factors, including track surface, wind speed, and humidity, likely contribute additional between-session variance not captured here. Finally, although ICCs identified the most reproducible indicators, the SEM and MDC95 values confirm that large between-session changes may still be required before an individual change can be confidently interpreted, particularly for HRR and speed-derived variables [21,22].

## 5. Conclusions

Routine short-term monitoring in French Standardbred trotters may benefit more from simple cardiovascular indicators such as cardiac cost and heart rate recovery, together with lower-threshold speed variables such as V150, than from relying exclusively on higher-threshold speed estimates. In the present study, CC during B1, HRR measured 60 s after both work blocks, and V150 showed the most favorable reproducibility and may therefore represent useful candidates for session-to-session monitoring under routine training conditions.

Among these, CC during B1 may be of practical interest. Derived from a submaximal effort, it is available before the horse reaches the highest training intensities of the session, and its reproducibility remained broadly stable after adjustment for ambient temperature across the conditions represented in this study. In addition, a between-session change exceeding 0.04 beats·m^−1^ is more likely to reflect a true physiological change rather than measurement error, suggesting that this indicator may provide useful mid-session information that complements post-session indicators. HRR and V150 may also complement this framework as post-session indicators of autonomic recovery and submaximal fitness, respectively.

By contrast, V180, mean block speeds, cardiac cost during B2, and recovery speed showed poor reproducibility and should be interpreted with caution for short-term individual monitoring. V200 showed only borderline reproducibility in this context but remains relevant for longer-term fitness assessment, as confirmed by its significant age-related increase in the full dataset.

These conclusions should be interpreted considering the single-yard design, the modest size of the short-term monitoring subset, and the substantial absolute measurement error observed even for the most reproducible variables. Accordingly, between-session changes should be interpreted cautiously at the individual level and considered meaningful only when they exceed the MDC95 thresholds reported here.

## Figures and Tables

**Table 1:** Descriptive characteristics of the full dataset and short-term monitoring subset.

Characteristic	Full Dataset	Short-Term Monitoring Subset
Horses, *n*	60	18
Sessions, *n*	483	126
Age, years	4.3 ± 1.9 (range 2.0–9.0)	4.5 ± 1.7 (range 2.0–7.5)
Female, *n*	30	11
Gelding, *n*	25	6
Stallion, *n*	5	1
Sessions per horse, median [IQR]	4.0 [2.0–9.0]	3.5 [3.0–7.8]
Blocks, *n*	—	36
Sessions per block, *n*	—	3.5 ± 0.8
Block duration, days, median [IQR]	—	11.0 [7.8–14.0]

The short-term monitoring subset was used for the reproducibility analyses. Follow-up blocks were defined as groups of consecutive monitored sessions within the same horse, with no more than 7 days between two successive monitored sessions. Block duration refers to the number of days elapsed between the first and last session within each follow-up block.

**Table 2:** Target speeds used in the monitored standardized training sessions according to age and training period.

Age (Training Period)	B1 Target Speed (km·h^−1^)	B2 Target Speed (km·h^−1^)
2 years old (0 to 4 months)	29.4	32.4
2 years old (4 to 12 months)	31.8	35.4
3 years old	33.6	37.8
4 years old	34.8	39.6

B1 and B2 correspond to the second and third stages of the training framework described by Demonceau and Auvinet (1992) [11].

**Table 3:** First-to-last session changes in selected cardiovascular and speed-related indicators within short-term follow-up blocks.

Variable	Unit	First Session, Mean	Last Session, Mean	Mean Change	*p* Value
V150	km·h^−1^	24.6	24.9	0.3 ± 4.2	0.70
V180	km·h^−1^	35.7	36.9	1.3 ± 3.4	0.04 *
V200	km·h^−1^	40.4	40.6	0.3 ± 3.1	0.62
Mean speed B1	km·h^−1^	35.7	35.8	0.1 ± 2.6	0.90
Mean speed B2	km·h^−1^	39.0	39.8	0.7 ± 2.1	0.046 *
Cardiac cost B1	beats·m^−1^	0.329	0.32	−0.005 ± 0.02	0.07
HR recovery 60 s after B1	bpm	128.8	127.4	−1.4 ± 11.2	0.46
Speed recovery 60 s after B1	km·h^−1^	8.4	9.2	0.8 ± 4.8	0.47 ^a^
Cardiac cost B2	beats·m^−1^	0.321	0.317	−0.005 ± 0.03	0.17 ^a^
HR recovery 60 s after B2	bpm	144.6	145.6	1.0 ± 11.1	0.58
Speed recovery 60 s after B2	km·h^−1^	13.4	13.0	−0.4 ± 4.9	0.61

Note: Values are presented as mean values at the first and last session of each follow-up block, together with mean change ± SD. *p*-values are from paired *t*-tests unless otherwise indicated. ^a^ Wilcoxon signed-rank test. * *p* < 0.05. V150, V180, V200: speed at a heart rate of 150, 180, and 200 beats·min^−1^, respectively; HR: heart rate; bpm: beats per minute; B1, B2: first and second work blocks, respectively; beats·m^−1^: beats per meter.

**Table 4:** Age-related associations for selected cardiovascular and speed-related indicators in the full dataset.

Variable	Age Estimate	SE	*p* Value
V150	0.18	0.15	0.22
V180	0.19	0.10	0.07
V200	0.40	0.10	<0.001
Cardiac cost B1	−0.005	0.001	<0.001
HR recovery 60 s after B1	−3.76	0.63	<0.001
Speed recovery 60 s after B1	−0.56	0.13	<0.001
Cardiac cost B2	−0.005	0.001	<0.001
HR recovery 60 s after B2	−4.65	0.58	<0.001
Speed recovery 60 s after B2	−0.40	0.12	<0.01

Note: Age estimate represents the fixed-effect estimate for age from the mixed-effects model, adjusted for sex and mean session temperature, with horse included as a random effect. SE: standard error; V150, V180, and V200, speed at a heart rate of 150, 180, and 200 beats·min^−1^, respectively; HR: heart rate; B1 and B2: first and second work blocks, respectively.

## Data Availability

The data presented in this study are available on request from the corresponding author.

## Supplementary Materials

The following supporting information can be downloaded at: https://www.mdpi.com/article/10.3390/ani16111598/s1, Table S1: (A): Descriptive characteristics of the monitoring subsets according to the maximum gap between sessions used to define short-term follow-up blocks; (B): Adjusted intra-block reproducibility of selected indicators according to the maximum gap between sessions used to define short-term follow-up blocks; Table S2: Intra-block reproducibility and absolute measurement error of the main adjusted indicators of the main cardiovascular and speed-related indicators in the 7-day monitoring subset, expressed as standard error of measurement (SEM) and minimal detectable change at the 95% confidence level (MDC95); Table S3: Descriptive statistics of the selected cardiovascular and speed-related indicators in the 7-day short-term monitoring subset; Table S4: Comparison of adjusted ICC estimates obtained with age and sex adjustment only versus additional adjustment for mean session temperature in the 7-day short-term monitoring subset.

## References

[1] Couroucé A. (1999). Field Exercise Testing for Assessing Fitness in French Standardbred Trotters. Vet. J..

[2] Dubreucq C., Chatard J.C., Courouce A., Auvinet B. (1995). Reproducibility of a Standardised Exercise Test for Standardbred Trotters under Field Conditions. Equine Vet. J..

[3] Couroucé A., Chrétien M., Valette J.P. (2002). Physiological Variables Measured under Field Conditions According to Age and State of Training in French Trotters. Equine Vet. J..

[4] Leleu C., Cotrel C., Courouce-Malblanc A. (2005). Relationships between Physiological Variables and Race Performance in French Standardbred Trotters. Vet. Rec..

[5] Navas De Solis C. (2019). Cardiovascular Response to Exercise and Training, Exercise Testing in Horses. Vet. Clin. N. Am. Equine Pract..

[6] Munsters C.C.B.M., Van Iwaarden A., Van Weeren R., Sloet Van Oldruitenborgh-Oosterbaan M.M. (2014). Exercise Testing in Warmblood Sport Horses under Field Conditions. Vet. J..

[7] Billat V.L., Palacin F., Correa M., Pycke J.-R. (2020). Pacing Strategy Affects the Sub-Elite Marathoner’s Cardiac Drift and Performance. Front. Psychol..

[8] Geor R.J., McCutcheon L.J., Ecker G.L., Lindinger M.I. (1995). Thermal and Cardiorespiratory Responses of Horses to Submaximal Exercise under Hot and Humid Conditions. Equine Vet. J..

[9] Geor R.J., McCutcheon L.J., Lindinger M.I. (1996). Adaptations to Daily Exercise in Hot and Humid Ambient Conditions in Trained Thoroughbred Horses. Equine Vet. J..

[10] Thomas D.P., Fregin G.F. (1990). Cardiorespiratory Drift during Exercise in the Horse. Equine Vet. J..

[11] Demonceau T., Auvinet B. (1992). Test d’effort de terrain pour trotteurs à l’entraînement: Réalisation pratique et premiers résultats. Proceedings of the 18ème Journée D’étude: Quoi de Neuf en Matière D’études et de Recherches sur le Cheval?.

[12] Kapteijn C.M., Frippiat T., Van Beckhoven C., Van Lith H.A., Endenburg N., Vermetten E., Rodenburg T.B. (2022). Measuring Heart Rate Variability Using a Heart Rate Monitor in Horses (Equus Caballus) during Groundwork. Front. Vet. Sci..

[13] Frippiat T., Van Beckhoven C., Moyse E., Art T. (2021). Accuracy of a Heart Rate Monitor for Calculating Heart Rate Variability Parameters in Exercising Horses. J. Equine Vet. Sci..

[14] Mott R., Dowell F., Evans N. (2021). Use of the Polar V800 and Actiheart 5 Heart Rate Monitors for the Assessment of Heart Rate Variability (HRV) in Horses. Appl. Anim. Behav. Sci..

[15] Akyildiz Z., Yildiz M., Clemente F.M. (2022). The Reliability and Accuracy of Polar Team Pro GPS Units. Proc. Inst. Mech. Eng. Part P J. Sports Eng. Technol..

[16] Sagiroglu İ., Akyildiz Z., Yildiz M., Clemente F.M. (2023). Validity and Reliability of Polar Team Pro GPS Units for Assessing Maximum Sprint Speed in Soccer Players. Proc. Inst. Mech. Eng. Part P J. Sports Eng. Technol..

[17] Ringmark S., Lindholm A., Hedenström U., Lindinger M., Dahlborn K., Kvart C., Jansson A. (2015). Reduced High Intensity Training Distance Had No Effect on VLa4 but Attenuated Heart Rate Response in 2-3-Year-Old Standardbred Horses. Acta Vet. Scand..

[18] Bates D., Mächler M., Bolker B., Walker S. (2015). Fitting Linear Mixed-Effects Models Using Lme4. J. Stat. Soft..

[19] Kuznetsova A., Brockhoff P.B., Christensen R.H.B. (2017). lmerTest Package: Tests in Linear Mixed Effects Models. J. Stat. Soft..

[20] Koo T.K., Li M.Y. (2016). A Guideline of Selecting and Reporting Intraclass Correlation Coefficients for Reliability Research. J. Chiropr. Med..

[21] De Vet H.C., Terwee C.B., Ostelo R.W., Beckerman H., Knol D.L., Bouter L.M. (2006). Minimal Changes in Health Status Questionnaires: Distinction between Minimally Detectable Change and Minimally Important Change. Health Qual. Life Outcomes.

[22] Dontje M.L., Dall P.M., Skelton D.A., Gill J.M.R., Chastin S.F.M., on behalf of the Seniors USP Team (2018). Reliability, Minimal Detectable Change and Responsiveness to Change: Indicators to Select the Best Method to Measure Sedentary Behaviour in Older Adults in Different Study Designs. PLoS ONE.

[23] Katz L.M. (2024). A Comparative Review of Heart Rate Recovery in the Human and Equine Athlete. UK-Vet Equine.

[24] Michael S., Graham K.S., Davis G.M. (2017). Cardiac Autonomic Responses during Exercise and Post-Exercise Recovery Using Heart Rate Variability and Systolic Time Intervals—A Review. Front. Physiol..

[25] Lo Feudo C.M., Stucchi L., Stancari G., Conturba B., Bozzola C., Zucca E., Ferrucci F. (2023). Evaluation of Fitness Parameters in Relation to Racing Results in 245 Standardbred Trotter Horses Submitted for Poor Performance Examination: A Retrospective Study. PLoS ONE.

[26] Vermeulen A.D., Evans D.L. (2006). Measurements of Fitness in Thoroughbred Racehorses Using Field Studies of Heart Rate and Velocity with a Global Positioning System. Equine Vet. J..

[27] Kingston J.K., Soppet G.M., Rogers C.W., Firth E.C. (2006). Use of a Global Positioning and Heart Rate Monitoring System to Assess Training Load in a Group of Thoroughbred Racehorses. Equine Vet. J..

[28] Schrurs C., Dubois G., Van Erck-Westergren E., Gardner D.S. (2024). Cardiovascular Fitness and Stride Acceleration in Race-Pace Workouts for the Prediction of Performance in Thoroughbreds. Animals.

