# Supplementary material for: Cardiac Cost During Submaximal Exercise as a Practical Monitoring Tool in French Standardbred Trotters: Short-Term Reproducibility of Non-Invasive Field-Derived Indicators
Source: Animals (Basel). 2026 May 24;16(11):1598. doi: 10.3390/ani16111598 (PMC13255733; doi:10.3390/ani16111598)
Supplement: Supplementary file 1 [file animals-16-01598-s001.zip › animals-4303231-supplementary.pdf]

Supplementary Table S1A Descriptive characteristics of the monitoring subsets according to the maximum gap between sessions used to define short-term follow-up blocks.

Characteristic	4-day threshold	7-day threshold	15-day threshold
Horses, n	12	18	28
Blocks, n	16	36	58
Sessions, n	53	126	243
Age, years	4.9 ± 1.7	4.5 ± 1.7	4.6 ± 1.7
Sessions per horse, median (IQR)	3.0 (3.0–4.5)	3.5 (3.0–7.8)	4.5 (3.0–12.0)
Sessions per block	3.3 ± 0.6	3.5 ± 0.8	4.2 ± 1.8
Block duration, days, median (IQR)	7.0 (7.0–7.8)	11.0 (7.8–14.0)	18.0 (14.0–26.5)
Female, n (%)	8 (66.7%)	11 (61.1%)	13 (46.4%)
Gelding, n (%)	4 (33.3%)	6 (33.3%)	13 (46.4%)
Stallion, n (%)	0 (0.0%)	1 (5.6%)	2 (7.1%)

Note: IQR: interquartile range.

Supplementary Table S1B Adjusted intra-block reproducibility of selected indicators according to the maximum gap between sessions used to define short-term follow-up blocks.

Variable	4-day threshold, adjusted ICC (95% CI)	7-day threshold, adjusted ICC (95% CI)	15-day threshold, adjusted ICC (95% CI)
V150	0.61 (0.27–0.80)	0.59 (0.39–0.73)	0.44 (0.29–0.57)
V180	0.03 (0.00–0.32)	0.17 (0.00–0.35)	0.16 (0.03–0.29)
V200	0.40 (0.05–0.65)	0.50 (0.28–0.66)	0.33 (0.17–0.46)
Mean speed B1	0.21 (0.00–0.51)	0.31 (0.09–0.48)	0.25 (0.09–0.38)
Mean speed B2	0.00 (0.00–0.28)	0.19 (0.00–0.38)	0.16 (0.03–0.28)
Cardiac cost B1	0.71 (0.38–0.86)	0.67 (0.48–0.78)	0.39 (0.23–0.52)
HR recovery 60 s after B1	0.48 (0.08–0.72)	0.60 (0.40–0.73)	0.45 (0.29–0.58)
Speed recovery 60 s after B1	0.00 (0.00–0.30)	0.07 (0.00–0.24)	0.06 (0.00–0.18)
Cardiac cost B2	0.29 (0.00–0.58)	0.22 (0.00–0.41)	0.20 (0.06–0.32)
HR recovery 60 s after B2	0.65 (0.33–0.82)	0.66 (0.47–0.78)	0.52 (0.37–0.64)
Speed recovery 60 s after B2	0.12 (0.00–0.41)	0.01 (0.00–0.18)	0.00 (0.00–0.10)

Note: Adjusted ICCs were estimated after adjustment for age, sex, and mean session temperature. ICC: intraclass correlation coefficient; CI: confidence interval; V150, V180, V200: speed at a heart rate of 150, 180, and 200 beats·min^{-1}, respectively; HR: heart rate; B1, B2: first and second work blocks, respectively.

Supplementary Table S2 Intra-block reproducibility and absolute measurement error of the main adjusted indicators of the main cardiovascular and speed-related indicators in the 7-day monitoring subset, expressed as standard error of measurement (SEM) and minimal detectable change at the 95% confidence level (MDC95).

Variable	Unit	Crude ICC (95% CI)	Adjusted ICC (95% CI)	SEM	MDC95
V150	km/h	0.62 (0.44–0.75)	0.59 (0.39–0.73)	2.40	6.64
V180	km/h	0.21 (0.01–0.38)	0.17 (0.00–0.35)	2.49	6.90
V200	km/h	0.50 (0.29–0.65)	0.50 (0.28–0.66)	1.86	5.16
Cardiac cost B1	beats/m	0.72 (0.56–0.82)	0.67 (0.48–0.78)	0.01	0.04
HR recovery 60 s after B1	beats/min	0.63 (0.44–0.76)	0.60 (0.40–0.73)	9.24	25.6
Cardiac cost B2	beats/m	0.36 (0.15–0.54)	0.22 (0.00–0.41)	0.02	0.05
HR recovery 60 s after B2	beats/min	0.71 (0.54–0.81)	0.66 (0.47–0.78)	7.81	21.6

Note: Adjusted ICCs were estimated after adjustment for age, sex, and mean temperature. ICC: intraclass correlation coefficient; CI: confidence interval; V150, V180, V200: speed at a heart rate of 150, 180, and 200 beats·min^{-1}, respectively; HR: heart rate; B1, B2: first and second work blocks, respectively; SEM: standard error of measurement; MDC95: minimal detectable change at the 95% confidence level.

Supplementary Table S3 Descriptive statistics of the selected cardiovascular and speed-related indicators in the 7-day short-term monitoring subset.

Variable	Unit	n	Mean ± SD	Median (IQR)
V150	km·h^{-1}	126	25.0 ± 3.93	25.1 [21.9–28.1]
V180	km·h^{-1}	126	36.1 ± 2.79	36.0 [34.6–38.2]
V200	km·h^{-1}	126	40.3 ± 2.64	40.3 [38.5–41.7]
Mean speed B1	km·h^{-1}	126	35.7 ± 2.24	36.0 [34.3–37.4]
Mean speed B2	km·h^{-1}	126	39.3 ± 1.85	39.4 [38.0–40.7]
Cardiac cost B1	beats·m^{-1}	126	0.32 ± 0.02	0.32 [0.31–0.34]
HR recovery 60 s after B1	bpm	126	127 ± 15	124 [118–132]
Speed recovery 60 s after B1	km·h^{-1}	126	8.9 ± 3.66	7.6 [6.8–9.7]
Cardiac cost B2	beats·m^{-1}	126	0.32 ± 0.03	0.32 [0.31–0.33]
HR recovery 60 s after B2	bpm	126	144 ± 14	142 [133–151]
Speed recovery 60 s after B2	km·h^{-1}	126	13.2 ± 4.14	13.5 [9.9–15.8]

Note: Descriptive statistics were calculated from all available observations in the 7-day short-term monitoring subset. Values are presented as mean ± SD and median (IQR). V150, V180, and V200, speed at a heart rate of 150, 180, and 200 beats·min^{-1}, respectively. HR, heart rate. B1 and B2, first and second work blocks, respectively.

Supplementary Table S4 Comparison of adjusted ICC estimates obtained with age and sex adjustment only versus additional adjustment for mean session temperature in the 7-day short-term monitoring subset.

**Variable**	**Adjusted ICC, age + sex**	**Adjusted ICC, age + sex + temperature**	**Difference**
V150	0.586	0.588	0.002
V180	0.161	0.167	0.006
V200	0.476	0.498	0.022
Mean speed B1	0.392	0.313	−0.079
Mean speed B2	0.231	0.192	−0.039
Cardiac cost B1	0.661	0.668	0.007
HR recovery 60 s after B1	0.601	0.603	0.002
Speed recovery 60 s after B1	0.054	0.066	0.012
Cardiac cost B2	0.214	0.218	0.004
HR recovery 60 s after B2	0.660	0.658	−0.002
Speed recovery 60 s after B2	0.095	0.010	−0.085

Note: ICCs were estimated from linear mixed-effects models within the 7-day short-term monitoring subset. The first model included age and sex as fixed effects, whereas the second additionally included mean session temperature. Difference was calculated as ICC adjusted for age, sex, and temperature minus ICC adjusted for age and sex only. ICC: intraclass correlation coefficient; V150, V180, V200: speed at a heart rate of 150, 180, and 200 beats·min^{-1}, respectively; HR: heart rate; B1, B2: first and second work blocks, respectively.
